# Improved homology-driven computational validation of protein-protein interactions motivated by the evolutionary gene duplication and divergence hypothesis

**DOI:** 10.1186/1471-2105-10-21

**Published:** 2009-01-19

**Authors:** Christian Frech, Michael Kommenda, Viktoria Dorfer, Thomas Kern, Helmut Hintner, Johann W Bauer, Kamil Önder

**Affiliations:** 1 Upper Austria University of Applied Sciences, Softwarepark 11, 4232 Hagenberg, Austria; 2 Paracelsus Medical Private University, Department of Dermatology, Müllner Hauptstraße 48, 5020 Salzburg, Austria; 3 Department of Cell Biology, University of Salzburg, Salzburg, Austria

## Abstract

**Background:**

Protein-protein interaction (PPI) data sets generated by high-throughput experiments are contaminated by large numbers of erroneous PPIs. Therefore, computational methods for PPI validation are necessary to improve the quality of such data sets. Against the background of the theory that most extant PPIs arose as a consequence of gene duplication, the sensitive search for homologous PPIs, i.e. for PPIs descending from a common ancestral PPI, should be a successful strategy for PPI validation.

**Results:**

To validate an experimentally observed PPI, we combine FASTA and PSI-BLAST to perform a sensitive sequence-based search for pairs of interacting homologous proteins within a large, integrated PPI database. A novel scoring scheme that incorporates both quality and quantity of all observed matches allows us (1) to consider also tentative paralogs and orthologs in this analysis and (2) to combine search results from more than one homology detection method. ROC curves illustrate the high efficacy of this approach and its improvement over other homology-based validation methods.

**Conclusion:**

New PPIs are primarily derived from preexisting PPIs and not invented *de novo*. Thus, the hallmark of true PPIs is the existence of homologous PPIs. The sensitive search for homologous PPIs within a large body of known PPIs is an efficient strategy to separate biologically relevant PPIs from the many spurious PPIs reported by high-throughput experiments.

## Background

Physical interactions between proteins, commonly referred to as protein-protein interactions (PPIs), occur at every level of cell function to elaborate the organism's phenotype. The study of PPIs is therefore of great interest and is helping to reveal basic molecular mechanisms of many diseases. High-throughput screening methods have given insight into hundreds of thousands of potential PPIs in several organisms. However, a major disadvantage of high-throughput approaches is their high rate of *false-positive *PPIs, i.e. erroneously reported PPIs that do not occur *in vivo *[1-7].

The development and implementation of computational methods for the validation of experimentally determined PPIs is therefore an important goal in bioinformatics today. Common approaches include determining intersections between different high-throughput PPI data sets [3], incorporating protein annotation data [5,8], analyzing expression profiles [4,9-12], investigating topological criteria of PPI networks [13-17], and inspecting patterns of co-evolution [18].

**Figure 1:**
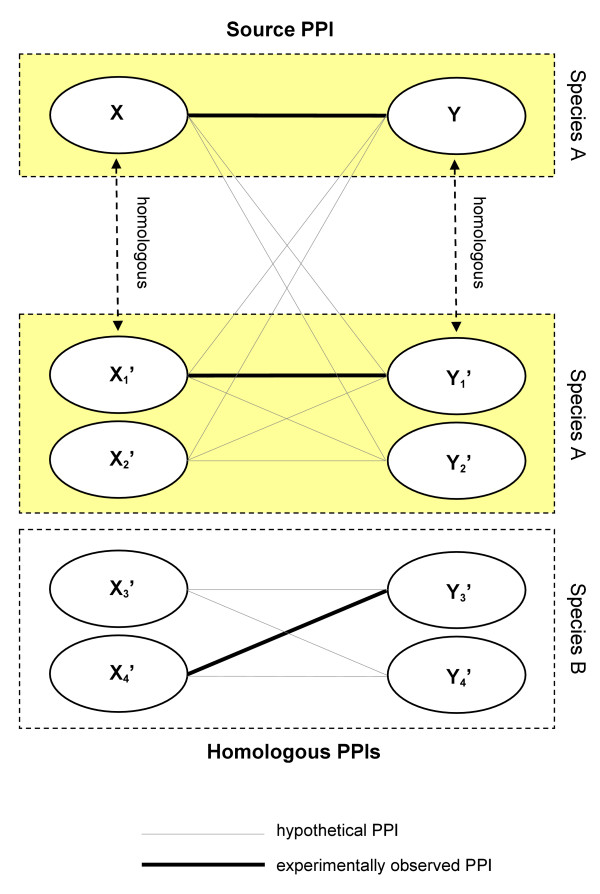
**Homology-Based PPI Validation**. Concept of homology-based PPI validation: based on an experimentally observed physical interaction between two proteins, X and Y (the questioned 'source' PPI), homologs of both proteins are identified, for example by local sequence alignments. These homologs include both paralogs from within the same species and orthologs from other species. An interaction between a homolog of X and a homolog of Y is called a 'homologous PPI'. If an experimentally observed homologous PPI is found (thick lines), confidence in the questioned source PPI increases.

Another, well established *in silico *technique to validate an experimentally determined PPI is to check if homologs of the interacting proteins also interact; if so, the confidence of this PPI is increased. The original *interolog *concept suggests to examine PPIs among functionally conserved *orthologs*, i.e. functionally conserved proteins in other species that evolved from a common ancestor [19,20]. However, large-scale application of this method for PPI validation is strongly hampered by limited coverage of most interactomes and by low numbers of known *bona fide *orthologs [21]. A first practical approach involved the inspection of PPIs among *paralogous *proteins, i.e. homologous proteins that evolved by gene duplication and are found within the same species [4]. Nevertheless, sensitivity remains a problem because in most organisms assured paralogs with known interactions are scarce. The strategy illustrated in Figure 1, which is followed in this paper, searches for homologous PPIs independent of species boundaries or functional constraints, which significantly increases the amount of PPI data usable for validation purposes (if not stated otherwise, the term 'homologous PPI' is understood as defined in Figure 1). Several papers applied this 'all-inclusive' approach to homology-based PPI validation [8,22,23]. Also techniques developed for PPI prediction, a relatively more well-studied bioinformatics problem, successfully utilized this idea, for example Brown *et al*. [24] or Jonsson *et al*. [25]. However, the focus of the present paper is not PPI prediction but the computational validation of experimentally determined PPIs.

Firstly, we draw the reader's attention to the duplication-divergence hypothesis of PPI evolution, i.e. the idea that extant PPIs primarily originate from gene duplications, the homologs diverging over time. If PPIs share common evolutionary ancestry, which is what this hypothesis suggests, then this ancestry reaches far into the evolutionary past. Consequently, homology-based PPI validation should investigate also diverged homologs and not only similar proteins.

Secondly, motivated by this idea, we propose an improved, sequence-based procedure for homology-based PPI validation. Unlike previously published, mostly binary validation schemes that deem a questioned PPI as biologically relevant as soon as a single homologous PPI is found, we follow a similar approach as Jonsson *et al*. [25] and compute a confidence score that takes into account both the quality and quantity of all identified homologous PPIs. The assignment of higher scores to high-quality hits and of lower scores to low-quality hits allows us to extend the search for homologous PPIs from reliable homologs to highly putative paralogs and orthologs with E-values up to 10. In addition to similar scoring schemes proposed before, we normalize and combine scores obtained from different homology search strategies.

Thirdly, we demonstrate the high efficacy of homology-based validation when carried out on large PPI data sets. A comprehensive data set of known physical binary PPIs from six PPI source databases is compiled, comprising 135,276 PPIs from 20 different organisms. This is, to the best of our knowledge, the largest collection of PPIs that has been used so far in this kind of analysis. Based on Receiver Operating Characteristic (ROC) curves it is shown that the new approach improves over previous methods for homology-based PPI validation.

## Results and discussion

### Duplication-Divergence Hypothesis of PPI Evolution

Gene duplication is a ubiquitous mechanism in molecular evolution and the principal source of biological innovation, producing new proteins and novel functional domains [26-30]. Here, we follow the idea that the duplication of genetic material coupled with subsequent divergence is also the dominant mechanism for the development of novel PPIs [31]. This hypothesis is supported by both theoretical models [32-34] and empirical evidence [35-40]. A brief review of papers supporting the duplication-divergence hypothesis can be found in Additional file 1.

**Figure 2:**
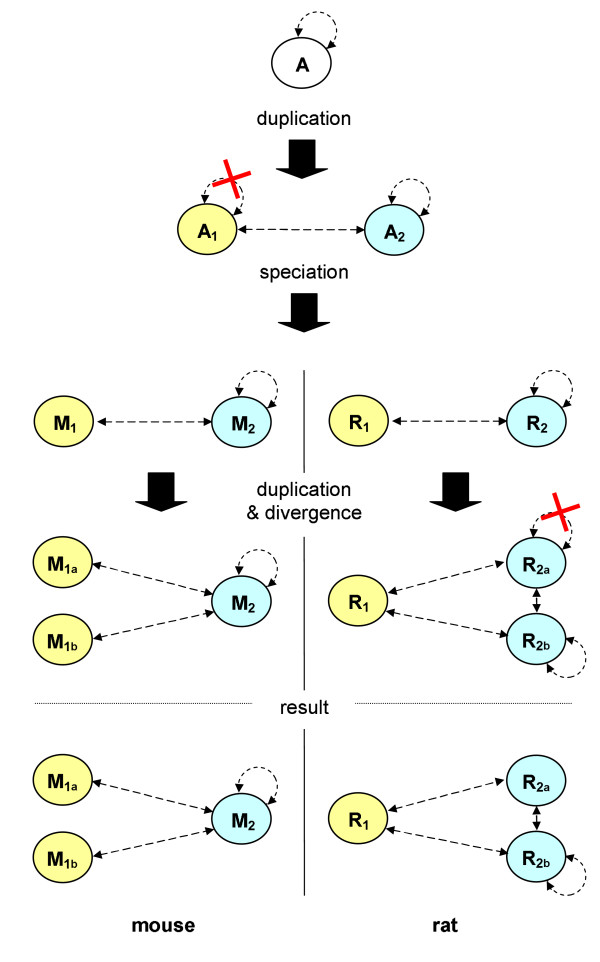
**Duplication-Divergence Model of PPI Evolution**. Simplified gene tree illustrating the emergence of new PPIs under the duplication-divergence model of PPI evolution. In an ancestral species, the gene encoding a self-interacting protein, A, is duplicated. From the resulting genes A_1 _and A_2_, A_1 _at some point loses its capability for self-interaction. Subsequent speciation forms the rat (R) and mouse (M) lineages, which evolve differently: in the mouse lineage, gene M_1 _is duplicated again, in the rat lineage R_2 _is duplicated. One of the R_2 _duplicates loses its capability for homodimerization due to deleterious mutations. Colors indicate the two groups of orthologous genes. Note that all depicted PPIs are homologous in the narrow sense of the word, because they share a common ancestor.

Duplication-divergence models of PPI evolution propose a simple and yet plausible idea of how evolution might have formed PPI networks over millions of years – by repeated duplication of interacting genes followed by their divergence. Figure 2 illustrates this idea.

### Implications of the Duplication-Divergence Model for Homology-Based PPI Validation

The duplication-divergence model of PPI evolution as shown in Figure 2 suggests that most biologically relevant PPIs descend from a common ancestral PPI, i.e. the PPIs are homologous to each other. This allows assessing the plausibility of an experimentally determined PPI as follows: for a true-positive PPI, one expects to see many homologous PPIs, whereas for a false-positive PPI this should be less likely. A sensitive search for homologous PPIs should thus, in principle, be able to filter out large numbers of false-positive PPIs from experimental PPI data sets while retaining the bulk of true-positive PPIs. However, both the incompleteness of today's PPI data sets and the fact that common ancestry is often elusive represent major practical obstacles along the way.

For assessing the validity of an experimentally predetermined PPI, also 'weak' homologous PPIs can be informative ('weak' in the sense of 'weak signal of homology'). Their incidence might support or contradict the idea that a questioned PPI evolved by duplication-divergence, which in turn can strengthen or weaken the position that a PPI is biologically relevant. However, the value of weak homologous PPIs for PPI *prediction *is limited: one cannot infer from a given pair of interacting proteins that very distant homologs interact as well. In the majority of cases this prediction would be simply wrong, because due to divergence most duplicated PPIs are eventually lost. PPIs *inferred *by homology are thus only trustworthy if protein similarity is high [41,42] or if these PPIs are supported by complementary data [24].

### Weak Homologous Interactions – Signal or Noise?

**Figure 3:**
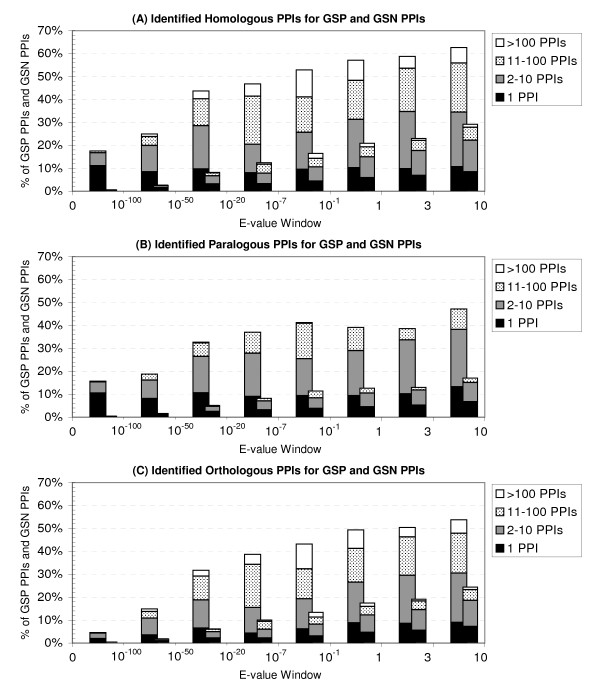
**Number of Homologous PPIs**. Percentage of GSP PPIs (*Combined *data set, left bars) and GSN PPIs (*Random *data set, right bars) with a certain number of homologous PPIs (A), paralogous PPIs (B), and orthologous PPIs (C). We investigated eight distinct E-value windows (x-axis) and used PSI-BLAST to determine the number of homologous PPIs within each of these windows (y-axis, numbers not cumulative). Each bar is composed of four distinct groups: the percentage of PPIs with a single identified homologous PPI, the percentage with 2 to 10 homologous PPIs, the percentage with 11 to 100 homologous PPIs, and the percentage with more than 100 identified homologous PPIs.

If the duplication-divergence model of PPI evolution is correct, the existence of weak homologous PPIs should be an observable characteristic of biologically relevant PPIs. We set out to test this hypothesis. For both Gold Standard Positive (GSP) and Gold Standard Negative (GSN) data sets, PSI-BLAST was used to search for homologous PPIs, and their distribution was determined within different E-value windows. Figure 3 shows the results. It reveals two important differences between GSP PPIs and GSN PPIs. Firstly, there is an increased probability for GSP PPIs to have at least one paralogous or orthologous PPI. Secondly, significantly more GSP PPIs than GSN PPIs have large numbers of paralogous and orthologous PPIs (>10). Most interestingly, both differences are observed up to high E-value windows.

Not surprisingly, the first characteristic, the existence of at least one homologous PPI, is a highly reliable signal for GSP PPIs when sequence similarity is high. For example, almost every fifth PPI taken out of the GSP data set (18%) has a homologous PPI with an E-value lower than 10^-100 ^(Figure 3A). By contrast, the existence of such high-quality homologs is extremely unlikely for a GSN PPI (0.25%). The signal remains intact with very low levels of sequence similarity: within the last E-value window (ranging from 3 to 10) the probability of observing a homologous PPI for a GSP PPI remains still twice as high (63%) as for a GSN PPI (30%).

The distribution of homologous PPIs reveals the second interesting characteristic of GSP PPIs: they tend to accumulate large numbers of homologous PPIs. According to Figure 3A, in all windows with E-values greater than 10^-20^, about 25% of GSP PPIs have more than 10 homologous PPIs; for GSN PPIs, this percentage never exceeds 8%. Thus, for many PPIs the existence of a large number of homologous PPIs is more conclusive than the existence of at least one homologous PPI, especially when sequence similarity is low: whereas about twice as many GSP PPIs than GSN PPIs have at least one homologous PPI within the last E-value window, almost four times as many GSP PPIs (21.4%) than GSN PPIs (5.8%) have between 10 and 100 homologs, and more than five times as many GSP PPIs (6.8%) than GSN PPIs (1.3%) have more than 100 homologs.

Both characteristics are observed independently of the fact whether only paralogous (Figure 3B) or only orthologous PPIs (Figure 3C) are investigated. For lower numbers of homologous PPIs, stronger signals on the paralogous data set are obtained than on the orthologous data set, which is consistent with the finding that PPIs seem to be more conserved within species than across species [41]. Interestingly, very large numbers (>100) of homologous PPIs are observed within the orthologous data set, most likely due to an increased number of gene duplications in higher eukaryotes.

We conclude that weak homologous PPIs are indeed an observable and distinguishing characteristic of biologically relevant PPIs, especially if they are observed in increased numbers. Consequently, weak homologous PPIs should be considered by homology-based PPI validation schemes.

### Overall Performance

**Figure 4:**
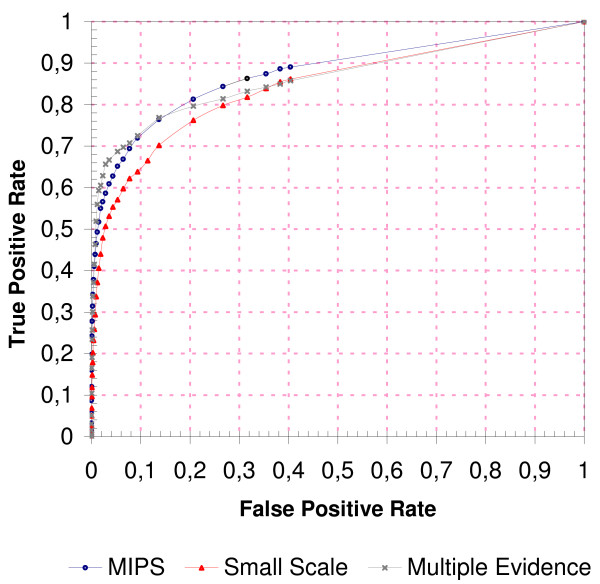
**Overall Performance**. Overall performance of the scoring scheme on the *MIPS*, the *Small Scale*, and the *Multiple Evidence *gold standard data sets. The *Random *data set served as the gold standard negative. Each data point of the curves corresponds to a pair of true-positive and false-positive rates, defined as the fraction of GSP PPIs and GSN PPIs that achieved a score above a sliding threshold. The threshold ranged from 1 to 10^-5 ^in this figure (values not shown). The area under the curve (AUC) is 84%, 86%, and 87% for the *Small Scale*, the *Multiple Evidence*, and the *MIPS *curve, respectively.

We devised a scoring scheme that incorporates the findings from Figure 3 (see Methods). The Receiver Operating Characteristic (ROC) curves in Figure 4 illustrate the overall performance of this scoring scheme for the *MIPS*, the *Small Scale *and the *Multiple Evidence *gold standard data sets. The y-axis shows the True-Positive Rate (TPR or *sensitivity*), i.e. the percentage of GSP PPIs that were correctly confirmed as biologically relevant. The x-axis represents the False-Positive Rate (FPR or 1-*specificity*), i.e. the percentage of GSN PPIs that were erroneously confirmed as biologically relevant. By varying the threshold of the score above which a PPI is confirmed as biologically relevant, different FPRs and TPRs are observed (a short introduction to ROC curves can be found in Additional file 1). For example, on the *MIPS *and the *Multiple Evidence *data sets, a TPR of more than 70% at an FPR of 10% is observed. An increased threshold results in a TPR of 80% at an FPR of 20% for the same two data sets. These values compare well to TPRs and FPRs reported by other, non-homology-based PPI validation techniques [11-14,18]. This underscores the high efficacy of homology-based PPI validation, especially when carried out on rich PPI data sets.

The *Small Scale *gold standard performs worst, which might reflect differences in the quality of the data sets. The *Multiple Evidence *gold standard can be considered of highest quality, because detection of a PPI with different experimental methods is a very reliable indicator of its existence [3]. Indeed, this data set achieves the best performance up to a TPR of 70%. The *MIPS *gold standard contains PPIs audited by human experts and is thus very trustworthy as well, although a slightly poorer performance is seen on this data set. PPIs of the *Small Scale *gold standard are not reviewed manually and thus its reliability might to a large degree reflect the quality of the automated text mining tools that are frequently used to extract them from the scientific literature. Because these tools are error-prone [43], the *Small Scale *gold standard might contain more spurious PPIs than the other two data sets.

Note that although the class distribution in the gold standard data sets is skewed, i.e. the *Random *GSN data set is about 50 times larger than each GSP data set, this does not affect the overall ROC curve [44]. In fact, we observed the same overall ROC curve on a balanced data set where the number of randomly chosen GSNs roughly equals the number of GSPs (data not shown). ROC curves as shown in Figure 4 are ideal to illustrate the overall performance of a classifier, but do not make suggestions about which specific score threshold should be applied to classify a PPI as true or false. This decision depends on the TPR and FPR one is willing to accept. Supplementary Figure 1 shows selected score thresholds and their associated TPRs and FPRs.

### Comparison of Homology-Based Validation Schemes

**Figure 5:**
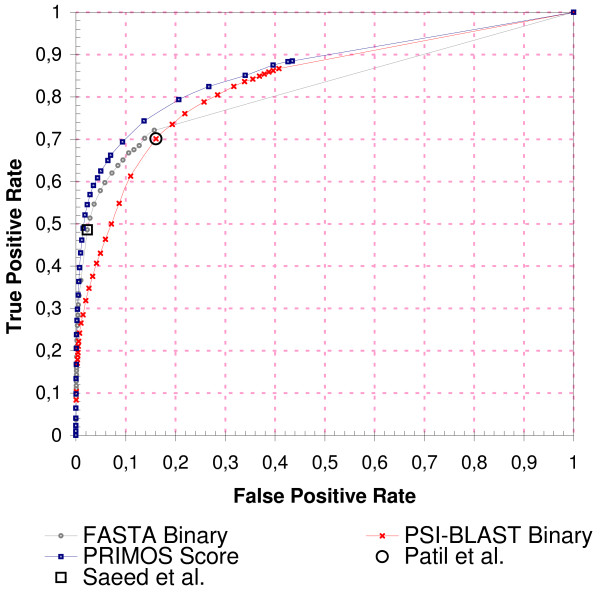
**Comparison of Homology-Based Validation Schemes**. Performance of the scoring scheme ('PRIMOS Score') in comparison to two conventional approaches (named 'FASTA Binary' and 'PSI-BLAST Binary' here), where a PPI is deemed as biologically relevant as soon as a single homologous PPI is found below a certain E-value. GSP PPIs comprised all PPIs from the *Combined *data set, the *Random *data set was used for the GSN PPIs. For the two binary schemes, we used FASTA and PSI-BLAST, respectively, to identify homologous PPIs and calculated the TPRs and FPRs as the fraction of GSP PPIs and GSN PPIs that had at least one homologous PPI below a sliding E-value threshold, ranging from 10^-300 ^to 10 in this figure. Black rectangle: parameter settings from Saeed and Deane [23]. Black circle: parameter setting used by Patil and Nakamura [8]. The area under the curve (AUC) is 82%, 83%, and 86% for the FASTA, PSI-BLAST, and PRIMOS curve, respectively.

Previous homology-based PPI validation methods involve simpler, binary selection processes in which a PPI is deemed to be biologically relevant as soon as a single homologous PPI is found [4,8,23]. Figure 5 shows a performance comparison with two of these methods. Note that this comparison does not include homology-based PPI *prediction *techniques, although these techniques are widely used. The reason is that these techniques have a different focus and generally incorporate also non-homology-based criteria, which makes a direct comparison difficult.

The FASTA-based binary validation scheme shows remarkably high specificity, even at high E-values. For example, inclusion of homologous PPIs with E-values between 10^-4 ^and 10 results in an increase of the TPR of almost 25% (from 48.5% to 72.1%), whereas the FPR grows only by 13.5% within the same interval, remaining below 16%. Considering the low sequence similarity and the probable existence of many spurious hits at E-values up to 10, this find is remarkable. By contrast, the PSI-BLAST-based binary validation scheme is less specific (even at low E-values), but much more sensitive: almost 87% of the GSP PPIs have at least one homologous PPI identified by PSI-BLAST (E-value ≤ 10). In addition to FASTA and PSI-BLAST, also BLAST was evaluated for homology detection (data not shown). In comparison to FASTA, no noticeable difference in performance was observed except for a slight decrease in maximum sensitivity (about 2% lower than with FASTA). Our scoring scheme, represented by the blue curve (squares), combines evidence from homologous PPIs found by FASTA and PSI-BLAST and clearly outperforms both individual binary validation schemes. For example, at a TPR of 70% it produces 4% fewer false-positives than the FASTA-based binary approach, and about 6% fewer false-positives than the PSI-BLAST-based binary validation scheme.

Previous homology-based validation schemes suggested different parameter settings. Saeed and Deane [23] used BLAST with an E-value up to 10^-4 ^to identify homologous PPIs and evaluated a TPR of 63% at an FPR of 7%. If this setting is transferred to FASTA and applied on our data sets, a TPR of 48.5% at an FPR of 2.3% is observed. Our scoring scheme, by contrast, produces only 1.5% false-positives at the same level of sensitivity (48.5%). The difference in FPR increases for higher levels of sensitivity, illustrating the additional value from incorporation of multiple methods for homology detection and from consideration of weak homologs. Patil and Nakamura [8] used PSI-BLAST with an E-value up to 10^-8 ^and reported a TPR of 89.7% at an FPR of 37.1% for their gold standards. On our data set the same parameter setting results in a significantly worse TPR of 70.1% at an FPR of 16.1%. Again, the scoring scheme outperforms and achieves a reduced FPR of 10% at the same level of sensitivity (70.1%). It is noteworthy that our exclusively sequence-based scoring scheme produces a superior ROC curve than Patil and Nakamura's Bayesian network approach, which incorporates three genomic features instead of one (sequence, structure and annotation information). This underscores again the potential efficacy of homology-based methods. No published PSI-BLAST parameters were found in the paper from Deane *et al*. [4], and thus the performance of this method was not assessed.

### Contribution of Weak Homologs

**Figure 6:**
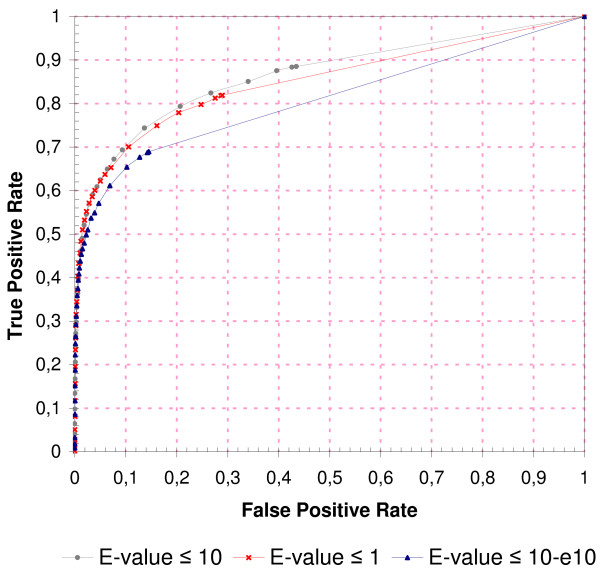
**Contribution of Weak Homologs**. Contribution of weak homologous PPIs to the overall classification performance of the scoring scheme. The gray ROC curve (squares) represents the original performance of the scoring scheme (considers all homologous PPIs with an E-value up to 10). The red ROC curve (crosses) illustrates the performance of the scoring scheme when only homologs with an E-value up to 1 are examined, and the blue curve (triangles) ignores all homologs with an E-value above 10^-10^. GSP PPIs were taken from the *Combined *data set, GSN PPIs comprised all PPIs from the *Random *data set. The area under the curve (AUC) is 80%, 85% and 86% for E-values 10^-10^, 1, and 10, respectively.

Is it actually beneficial to include homologous PPIs with high E-values (>1) for PPI validation, i.e. do weak homologs indeed contribute positively in terms of increased sensitivity and/or increased specificity? To answer this question, the classification performance of the scoring scheme with and without the inclusion of weak homologs was determined. Figure 6 shows the results.

The inclusion of weak homologous PPIs contributes positively to the overall classification performance. For example, the restriction of the analysis to homologous PPIs with an E-value below 10^-10 ^results in a maximum TPR of 69% at an FPR of 14.5%. When homologs with E-values up to 1 are considered, the same sensitivity is achieved at a significantly reduced FPR of 10%. Another increase of the E-value threshold up to 10 leads to a further reduction of the FPR by 1%.

Note that a similar effect cannot be observed for the classic, binary validation schemes, where less stringent E-value thresholds increase sensitivity but decrease specificity (Figure 5). This emphasizes the value of the scoring approach: it finds evidence for biologically relevant PPIs among weaker homologs without the compromise of an increased rate of false-positives.

Although the additional benefit resulting from the inclusion of weak homologs with an E-value above 1 is rather low on this data set, we expect it to increase for data sets where high-quality homologs are not at hand. Yeast is comparably well investigated, with approximately 50% of its estimated 40,000 to 75,000 PPIs known [45]. As a consequence, most of its biologically relevant PPIs have homologous PPIs among high-quality paralogs, and matches among weak homologs add little extra value to the overall score. This situation is different from most other organisms where interactome coverage is far below 50% and where weak paralogs and orthologs are often the only possibility to validate a questioned PPI.

## Conclusion

Knowledge of PPIs is key to understanding cell function. Although experimental high-throughput PPI detection techniques are now making it possible to catalogue all PPIs of a cell, the notoriously high error rates of these methods are a major obstacle to achieving this ambitious goal. Computational methods that can efficiently separate the PPI wheat from the chaff are therefore highly desirable.

We think that recent insights into the evolution of PPIs, in particular the duplication-divergence hypothesis, might be crucial to this endeavor. Nature is a tinkerer, not an inventor [46]. For PPIs this means that new PPIs are primarily derived from preexisting PPIs rather than invented *de novo*. Consequently, most true-positive PPIs must have homologous PPIs, not only among highly similar proteins, but also among distantly related proteins. This important characteristic of biologically relevant PPIs should, in principle, allow successful discrimination between true and false PPIs.

In light of this consideration, homology-based validation techniques seem promising, but have not gained much attention so far. Literature searches revealed only five papers that proposed a homology-based technique to validate experimental PPIs on a large scale, only three of which presented a critical performance assessment. Presumably this reflects the fact that homology-based validation requires having at hand a set of PPIs among homologous proteins, when few such PPIs have been known. However, with more and more PPIs now being reported from high-throughput experiments, this limitation is no longer factor.

In this paper, we assembled a large PPI data set to reassess the performance of homology-based PPI validation. It was shown that the classic, binary validation technique is efficient on such data sets, but can be further improved by using multiple methods for homology detection and more remote homologs to complement close homologs.

We expect the findings to be most relevant in situations where interactions among assured paralogs or orthologs are not at hand and thus traditional homology-based validation is not an option. Existing PPI databases could use the proposed method to reduce their number of false-positives without losing too many true-positives, especially within well explored model organisms. Other prospective applications include the elucidation of physically interacting proteins from known protein complexes, or the validation of *in silico *predicted PPIs in cases where homology was not used as a criterion for prediction in advance. Prospective improvements may involve more sophisticated methods for homology detection (e.g. Profile-HMMs), identification of PPI-mediating protein features (e.g. interacting domains) prior to homology detection to refine the selection of homologs, and an assessment of the statistical significance (P-values) of computed scores to obtain an intuitive measure of a PPI's validity.

## Methods

### Database Search for Homologous PPIs

The modular architecture of proteins implies that a protein has not just one distinct evolutionary trajectory, but one for each biological feature it contains [47]. Since in general PPI-mediating features (e.g. the domains) are unknown, one cannot selectively examine only trajectories of relevance. One possibility is to examine evolutionary trajectories of all protein features, but this comes with an increased risk of detecting PPIs that are not truly homologous. Also, if one wishes to include weak paralogs and orthologs in the analysis to capture gene duplication events that happened long time ago, methods for homology detection become inaccurate and produce spurious hits – another source of false homologous PPIs. To maximize both sensitivity and specificity despite these difficulties, we opt for a large-scale, sequence-based screening procedure in combination with a scoring scheme. Given an experimentally determined PPI, both FASTA [48] and PSI-BLAST [49] are used to search for homologous PPIs. FASTA supplies more reliable results for closely related proteins, while PSI-BLAST is more sensitive for remote relationships [50]. To further increase the sensitivity of the method, local sequence similarities with E-values up to 10 are considered. This produces many spurious hits, and thus traditional homology-based PPI validation techniques that simply check for the existence of a single homologous PPI become misleading (compare Figure 3). We therefore follow a similar approach as Jonsson *et al*. [25] and apply a scoring scheme that weighs each match according to its sequence similarity: low E-values score high, and high E-values score low. Thus high scores can result from few high-quality hits but also from numerous low-quality hits.

Homologous PPIs are searched within a subset of the Protein Interaction and Molecule Search (PRIMOS) database , release BETA-2.7/2007–04 [51]. This subset consists of 135,276 redundancy-removed, physical binary PPIs between 42,288 proteins from 20 organisms, imported from six primary PPI databases [52-57] (see Additional file 1). We used both FASTA (fasta34.exe, v3.4) and PSI-BLAST (blastpgp.exe, v2.2.16) to determine homologs for all proteins of our gold standard data sets. The search space for homologous proteins was restricted to the set of 42,288 proteins with known PPIs. According to Figure 1, we considered a homologous PPI as an interaction found between a pair of homologous proteins. E-value thresholds for both programs were set to 10, the *ktup *parameter of FASTA was set to 1, and the number of iterations for PSI-BLAST was set to 10. All other program parameters were left default.

### Gold Standard Data Sets

Four gold standard positive (GSP) data sets and one gold standard negative (GSN) data set with PPIs from *Saccharomyces cerevisiae *are used for performance assessment. Yeast is relatively well-studied, which allows being rather stringent in the selection of the GSP data sets. In addition, yeast has already been used numerous times in similar studies, which eases the comparison with previous results.

The *MIPS *GSP data set comprises 1,541 physical binary PPIs obtained from the Comprehensive Yeast Genome Database (CYGD) [57]. This database is considered as a high-quality resource for yeast PPIs and is frequently used as a gold standard reference set. PPIs reported by high-throughput experiments are excluded from this data set (see Additional file 1). The *Multiple Evidence *GSP data set consists of 393 PPIs reported by at least two experimental methods and in at least two different publications. As an additional criterion, only publications imported from one PPI database are considered. For example, if a PPI is reported from a publication contained in DIP and MINT, it will be excluded from the data set. If DIP is the only source database for this PPI, the PPI will be included. In an integrated dataset compiled from multiple source data sets this procedure reduces the risk that duplicate PPIs are regarded as homologous. The *Small Scale *data set consists of yeast PPIs reported by 'small-scale' experiments and contains 902 PPIs. Only PPIs of publications with up to three reported PPIs are considered. To minimize the risk of duplicate PPIs, publications imported from more than one primary PPI database are excluded (same procedure as for the *Multiple Evidence *GSP). The three GSP data sets overlap only to a low degree: just 8 PPIs are common to all three data sets, 25 between *MIPS *and *Small Scale*, 86 between *Small Scale *and *Multiple Evidence*, and 10 between *MIPS *and *Multiple Evidence*. The *Combined *GSP data set contains all PPIs from the previous three GSP data sets (2,723 PPIs in total).

The *Random *GSN PPI data set was generated by randomly selecting 50,000 protein pairs out of 7,058 yeast proteins (UniProt [58] release 10.0, downloaded on March 29, 2007) that were not found interacting within the PRIMOS database. A randomly selected data set is not completely free of real PPIs, but has no selection bias, for example towards protein pairs with different molecular functions [23]. The amount of real PPIs within such a randomly selected GSN data set should be generally low at about 0.25% [59].

### Scoring Scheme

The score S(*a*, *b*) of a queried interaction between two proteins *a *and *b *is defined as

(1) $$\text{S}(a,b)=\sum_{o\in O}\sum_{\begin{array}{c} (h_{a},h_{b})\in \\ {\text{H}}_{\text{a}}(o)\times {\text{H}}_{\text{b}}(o) \end{array}}\{\begin{array}{ll} \text{sim}(h_{a})\text{ sim}(h_{b}) & h_{a}\text{ interacts with }h_{b}\\ 0 & \text{otherwise} \end{array}$$

where *O *is the set of organisms with known experimental PPIs in the PRIMOS database, H_*a*_(*o*) and H_*b*_(*o*) denote the sets of proteins from organism *o *that are homologous to protein *a *and *b*, respectively. If there is experimental evidence for an interaction between homolog *h*_*a *_and homolog *h*_*b *_in the PRIMOS database, a score proportional to their sequence similarity is added to an overall sum.

Note that the computation of S(*a*, *b*) excludes homologous PPIs where the two proteins are from different organisms. Homologous PPIs from the same organism with one protein identical to one of the source PPI proteins are allowed. In this case, the E-value of the identical protein is assumed to be 0. Furthermore, if two identical homologous PPIs are found in an organism, i.e. two pairs $\left(h_{a_{1}},h_{b_{1}}\right)$ and $\left(h_{a_{2}},h_{b_{2}}\right)$ where $h_{a_{1}}=h_{b_{2}}$ and $h_{b_{1}}=h_{a_{2}}$, then only the homologous PPI with the lower E-value is considered. The other PPI is ignored. The E-value of a homologous PPI (*h*_*a*_, *h*_*b*_) is defined as max(evalue(*h*_*a*_), evalue(*h*_*b*_)). The similarity measure sim(*x*) of a homologous protein *x *is defined as

(2) $$\text{sim}(x)=\{\begin{array}{ll} 300 & \text{evalue}(x)=0\\ -\log_{10}\left(\frac{\text{evalue}(x)}{100}\right) & \text{otherwise} \end{array}$$

where evalue(*x*) is the E-value of homolog *x *reported by FASTA and PSI-BLAST, respectively (note that FASTA and PSI-BLAST scores are computed independently, see below). For each pair of interacting homologs, the scoring scheme basically extracts the positive exponent of the two reported E-values (-log_10_) and multiplies these exponents to get a joint similarity measure proportional to the similarity of both homologs. The total score is then the sum over all pairs of interacting homologs. Since a maximum E-value of 10 is allowed, division by 100 ensures that the negative logarithm is positive over the full range of possible E-values. The logarithm of zero is undefined, so E-values of zero are assigned the negative logarithm of roughly the smallest reported E-value greater than zero (10^-300^). This scoring scheme is similar to those proposed by Jonsson *et al*. [25], but uses more interpretable E-values instead of bit scores and puts more weight on the individual similarities of the two proteins (product of logarithms instead of logarithm of products). We found this weighing scheme important for rewarding high-quality hits where both homologs exhibit a high-degree of similarity, in which case the PPI in question is almost always true [23]. In addition, the score is then normalized, so that individual scores from different search strategies can be compared and combined.

(3) $${\text{S}}_{\text{norm}}(a,b)=\frac{\text{S}(a,b)}{{\text{S}}_{\max}(a,b)}$$

where S_max_(*a*, *b*) is defined as S(*a*, *b*) with all *h*_*a *_assumed as interacting with all *h*_*b*_. This scales the score to values ranging from 0 (minimum score) to 1 (maximum score).

Two normalized scores are computed independently, one with the homologs identified by FASTA and one with the homologs identified by PSI-BLAST. The final score is defined as the arithmetic mean of both normalized scores:

(4) $${\text{S}}_{\text{final}}(a,b)=\frac{{\text{S}}_{\text{norm}}^{\text{FASTA}}(a,b)+{\text{S}}_{\text{norm}}^{\text{PSIBLAST}}(a,b)}{2}$$

## Authors' contributions

CF conceived the method as well as its biological motivation, designed and conducted data analysis, and drafted the manuscript. MK and VD provided the data for analysis, were involved in many fruitful discussions, and revised the draft manuscript. TK supported in algorithm design and coordinated the project. HH and JB piloted the underlying PRIMOS system and contributed with biomedical knowhow. KÖ initiated the project, contributed with ideas throughout development, and revised the draft manuscript.

## Supplementary Material

Additional file 1 **Supplementary information.** File with supplementary information referenced in the main document. Click here for file
