# Supplementary material for: Improved homology-driven computational validation of protein-protein interactions motivated by the evolutionary gene duplication and divergence hypothesis
Source: BMC Bioinformatics. 2009 Jan 19;10:21. doi: 10.1186/1471-2105-10-21 (PMC2637843; doi:10.1186/1471-2105-10-21)
Supplement: Additional file 1 — Supplementary information. File with supplementary information referenced in the main document. [file 1471-2105-10-21-S1.pdf]

Additional file 1

Support for the Duplication-Divergence Hypothesis

Like social networks, computer networks or neural networks, biological networks have a *scale-free* topology (Jeong *et al.*, 2000; Yook *et al.*, 2004). This topological property generally develops in networks that grow over time and in which new nodes become preferentially attached to nodes that already have many connections, an effect known as *preferential attachment* (Barabasi and Albert, 1999). In PPI networks, preferential attachment might result from genetic duplications, whereby interacting proteins gain new PPIs over time through duplication of existing interaction partners (Vázquez *et al.*, 2003). Although in the course of evolution most duplicated proteins and PPIs are eventually lost due to the accumulation of deleterious mutations, many of them can be retained due to the important effect known as *sub-functionalization* (Prince and Pickett, 2002).

Computer simulations investigated if the combined effect of repeated genetic duplications and PPI losses due to subsequent divergence produces similar PPI networks as observed today. These models show that this is indeed the case, without the need to introduce new PPIs between previously non-interacting proteins (Vázquez *et al.*, 2003; Pastor-Satorras *et al.*, 2003).

Besides these theoretical models, also empirical studies produce more and more evidence that simple *duplication-divergence models* capture the essence of PPI evolution. In 2003, Eisenberg and Levanon investigated *S. cerevisiae* to clarify whether or not preferential attachment played a role in the growth of its PPI network (Eisenberg and Levanon, 2003). In a cross-genome comparison, they showed, "(a) the older a protein, the better connected it is, and (b) the number of interactions a protein gains during its evolution is proportional to its connectivity." They concluded that preferential attachment governs the protein network evolution. Light *et al.* analyzed the connectivity of the enzymes of *E. coli* to explain the scale-freeness of metabolic networks (Light *et al.*, 2005). In this work, they also found indications of preferential attachment. They argued, "a possible biological explanation for preferential attachment growth of metabolic networks is that novel enzymes created through gene duplication maintain some of the compounds involved in the original reaction, throughout its future evolution." This supports the idea of sub-functionalization mentioned earlier. Also gene-regulatory interaction networks have been investigated to find out if gene duplications played a major role in their development. By combining phylogenetic, proteomic and structural information, Amoutzias *et al.* elucidated the evolutionary driving forces for the gene-regulatory interaction networks of basic helix-loop-helix transcription factors (Amoutzias *et al.*, 2004). They inferred,

> "... recurrent events of single-gene duplication and domain rearrangement repeatedly gave rise to distinct networks with almost identical hub-based topologies, and multiple activators and repressors. We thus provide the first empirical evidence for scale-free protein networks emerging through single-gene duplications, the dominant importance of molecular modularity in the bottom-up construction of complex biological entities, and the convergent evolution of networks."

Another work focused on gene-regulatory interaction networks in *E. coli* and *S. cerevisiae* (Teichmann and Babu, 2004). From previous investigations it was already known that duplication of transcription factor genes followed by inheritance of interaction has contributed considerably to the growth of the regulatory network, with more than two-thirds of *E. coli* (77%) and *S. cerevisiae* (69%) transcription factors having at least one interaction in common with their duplicates. Teichmann and Babu found,

> "in both organisms, only a small fraction (10%) of the interactions evolved by innovation, consisting of transcription factors and target genes without

homologs. Almost 90% of the interactions evolved by duplication of either a transcription factor or a target gene."

van Noort *et al*. investigated the gene co-expression network in *S. cerevisiae*, in which genes are linked when they are coregulated (van Noort *et al*., 2004). They derived a simple duplication-divergence model for its evolution based on the observation that there is a positive correlation between the sequence similarity of paralogs and their probability of co-expression or sharing of transcription factor binding sites. They conclude that their model "reproduces the scale-free, small-world architecture of the coregulation network and the homology relations between coregulated genes without the need for selection either at the level of the network structure or at the level of gene regulation." Although this study is based on data from a completely different experimental technique, again the resulting protein network could be fully explained by two simple evolutionary events: gene duplication with subsequent divergence. Pereira-Leal and Teichmann emphasized the importance of duplication with subsequent divergence in the evolution of protein complexes (Pereira-Leal and Teichmann, 2005). They observed, "at least 6%-20% of the protein complexes have strong similarity to other complexes; thus a considerable fraction has evolved by duplication." In a second work, they studied protein complexes in *S. cerevisiae*, complexes of known three-dimensional structure in the Protein Data Bank and clusters of pairwise protein interactions in the networks of several organisms (Pereira-Leal *et al*., 2007). They found, "duplication of homomeric interactions, a large class of protein interactions, frequently results in the formation of complexes of paralogous proteins. This route is a common mechanism for the evolution of complexes and clusters of protein interactions." Interestingly, Ispolatov *et al.* (2005) found that homodimers on average have twice as many interaction partners than non-self-interacting proteins, suggesting that most of the interactions between paralogs are actually inherited from ancestral homodimeric proteins rather than established *de novo* after duplication. In fact, homodimers might well have played a crucial role in PPI evolution, since they have an increased affinity for self-interaction due to structural complementarity (Lukatsky *et al.*, 2007; Monod *et al.*, 1965), and could thus once have provided the 'raw material' for the evolution of larger, highly complex PPI networks.

MIPS Gold Standard Data Set

Our *MIPS* GSP data set was obtained from the Comprehensive Yeast Genome Database (CYGD)[1] (Güldener *et al.*, 2005). Excluded PubMed IDs (high-throughput experiments) were 10688190, 10655498, 11283351, 11743205, 11743162, 11087867, 11489916, 10900456, and 14764870.

Database Search for Homologous PPIs

For all our gold standard PPIs we searched for homologous PPIs within a subset of the Protein Interaction and Molecule Search (PRIMOS) database[2] (release BETA-2.7/2007-04). PRIMOS is an integrated PPI resource that collects PPI data from several sources, including BIND (Bader *et al.*, 2001) [72,656 PPIs], DIP (Salwinski *et al.*, 2004) [53,822 PPIs], HPRD (Peri *et al.*, 2003) [29,845 PPIs], IntAct (Hermjakob *et al.*, 2004) [66,751 PPIs], MINT (Chatr-aryamontri *et al.*, 2007) [87,884 PPIs], and MIPS (Güldener *et al.*, 2006) [14,208 PPIs]. In total, this yielded a pool of 325,166 imported binary physical PPIs. Binary PPI data that was explicitly declared as being derived from protein complex data was excluded. The total number of imported binary PPIs was further reduced by PPIs with interaction type "colocalization" (668 PPIs) or "genetic interaction" (3 PPIs). Finally, all PPIs between proteins that showed 100% sequence similarity to another pair of interacting proteins were merged, resulting in 135,276 redundancy-removed, physical binary PPIs between 42,288 proteins.

---

[1] ftp://ftpmips.gsf.de/yeast/PPI, downloaded on March 13, 2007

[2] http://primos.fh-hagenberg.at/

We used both FASTA (fasta34.exe, v3.4) and PSI-BLAST (blastpgp.exe, v2.2.16) to determine homologs for all proteins contained in our PPI gold standard data sets (see Gold Standard Data Sets). E-value thresholds for both programs were set to 10, the *ktup* parameter of FASTA was set to 1, and the number of iterations for PSI-BLAST was set to 10. All other program parameters were left default. Our search resulted in 487,008 homology assignments found by FASTA and 2,797,323 homology assignments determined by PSI-BLAST.

In a final filter step we excluded homologous PPIs from organisms with less than 100 PPIs. Due to codon bias and thus questionable search results homologous PPIs from *Plasmodium falciparum* were excluded from our analysis as well. Supplementary Table 1 lists finally considered organisms as well as the number of PPIs and the number of interacting proteins in each organism.

**Organism**	**PPIs**	**Proteins**
Homo sapiens	39809	12017
Drosophila melanogaster	37069	9820
Saccharomyces cerevisiae	34704	6190
Caenorhabditis elegans	7426	3699
Mus musculus	4734	3605
Escherichia coli	2776	1826
Escherichia coli O157:H7	1835	487
Arabidopsis thaliana	1623	811
Rattus norvegicus	1422	1069
Helicobacter pylori 26695	1382	700
Helicobacter pylori	829	596
Bos Taurus	363	279
Oryza sativa	270	256
Schizosaccharomyces pombe	257	261
Human herpesvirus 3	141	51
Xenopus laevis	141	146
Thermus thermophilus	135	124
Gallus gallus	130	130
Rickettsia sibirica 246	116	98
Bacillus subtilis	114	123

Supplementary Table 1: PRIMOS database subset used for PPI validation.

Background: Binary Classification and Receiver Operator Characteristics (ROC)

A *binary classifier* is a mapping of instances to two possible outcomes. For PPI validation, these outcomes are either (1) a PPI is predicted to be *true* or (2) a PPI is predicted to be *false*. There are four possible outcomes of this classification problem:

1) A PPI is predicted to be true and it is actually true;
then it is called a *true-positive* (TP).
2) A PPI is predicted to be true, but it is actually false;
then it is called a *false-positive* (FP).
3) A PPI is predicted to be false and it is actually false;
this is called a *true-negative* (TN).
4) A PPI is predicted to be false, but it is actually true;
then this is a *false-negative* (FN).

Two important measures of a binary classifier are the *True-Positive Rate* (TPR) and the *False-Positive Rate* (FPR).

$$TPR = \frac{TP}{TP + FN} = \text{sensitivity}$$

$$FPR = \frac{FP}{FP + TN} = (1 - \text{specificity})$$

The TPR is the fraction of correctly classified positives among all positive instances, and the FPR is the fraction of the incorrectly classified negatives among all negative instances. These two measures essentially capture the information of all four possible outcomes of a binary classifier. Note that the TPR is equivalent to *sensitivity*, and the FPR is equal to 1 − *specificity*.

One possibility to illustrate the overall performance of a binary classifier is to create a *Receiver Operator Characteristics* (ROC) curve. In signal detection theory, a ROC or ROC curve is a graphical plot of the FPR (x-axis) vs. TPR (y-axis). Each point in a ROC curve represents a pair of TPR and FPR values obtained by applying a different numerical threshold to the classifier (in our case, this classifier is the PPI score as introduced in the Methods section). The best possible prediction method would yield a point in the upper left corner of the ROC diagram, representing 100% sensitivity (all true-positives are found) and 100% specificity (all true-negatives are found). A completely random guess would give a point along a diagonal line (the line of no-discrimination), from the left bottom to the top right corners. In practice, classification performance lies somewhere in-between these two extremes. The closer the curve fits to the upper left corner of the diagram the better. For a more detailed introduction to ROC curves refer to the literature, for example Fawcett (2004).

Distribution of Scores

ROC curves as shown in the Figures 4, 5, and 6 are very useful to illustrate the overall performance of a classification method but do not explicitly convey information about which score threshold was actually used to achieve a certain TPR and FPR. Supplementary Figure 1 addresses this question.

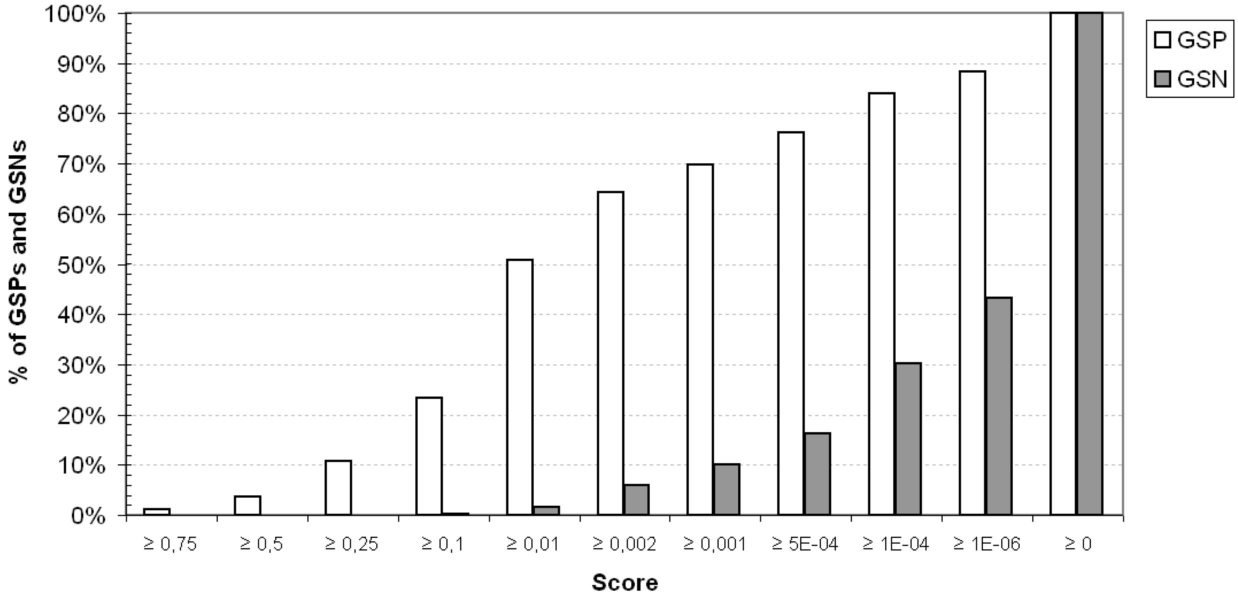

Supplementary Figure 1. Distribution of scores within the GSP and GSN data set. The x-axis depicts different score thresholds between 0 and 1, and the y-axis shows the percentage of GSP PPIs (white bars) and GSN PPIs (grey bars), respectively, that achieved a score equal or above this threshold. GSP PPIs comprised all PPIs of the *Combined* data sets (2,723 PPIs in total). GSN PPIs were 50,000 PPIs taken from our *Random* data set.

Bibliography

Amoutzias, G. D., Robertson, D. L., Oliver, S. G., and Bornberg-Bauer, E. (2004). Convergent evolution of gene networks by single-gene duplications in higher eukaryotes. EMBO Rep, 5(3), 274–279.

Bader, G. D., Donaldson, I., Wolting, C., Ouellette, B. F., Pawson, T., and Hogue, C.W. (2001). Bind–the biomolecular interaction network database. Nucleic Acids Res, 29(1):242–245.

Barabasi and Albert (1999). Emergence of scaling in random networks. Science, 286(5439), 509–512.

Ben-Hur, A. and Noble, W. S. (2006). Choosing negative examples for the prediction of protein-protein interactions. BMC Bioinformatics, 7 Suppl 1:S2.

Chatr-aryamontri, A., Ceol, A., Palazzi, L. M., Nardelli, G., Schneider, M. V., Castagnoli, L., and Cesareni, G. (2007). Mint: the molecular interaction database. Nucleic Acids Res, 35(Database issue), D572–D574.

Deng, M., Sun, F., and Chen, T. (2003). Assessment of the reliability of protein-protein interactions and protein function prediction. Pac Symp Biocomput, pages 140–151.

Eisenberg, E. and Levanon, E. Y. (2003). Preferential attachment in the protein network evolution. Phys Rev Lett, 91(13), 138701.

Fawcett, T. (2004). ROC Graphs: Notes and Practical Considerations for Researchers, 2004. Submitted to Machine Learning.

Goldberg, D. S. and Roth, F. P. (2003). Assessing experimentally derived interactions in a small world. Proc Natl Acad Sci U S A, 100(8), 4372–4376.

Güldener, U., Münsterkötter, M., Oesterheld, M., Pagel, P., Ruepp, A., Mewes, H., and Stümpflen, V. (2006). Mpact: the mips protein interaction resource on yeast. Nucleic Acids Res, 34(Database issue):D436–D441.

Hermjakob, H.,Montecchi-Palazzi, L., Lewington, C.,Mudali, S., Kerrien, S., Orchard, S., Vingron, M., Roechert, B., Roepstorff, P., Valencia, A.,Margalit, H., Armstrong, J., Bairoch, A., Cesareni, G., Sherman, D., and Apweiler, R. (2004). Intact: an open source molecular interaction database. Nucleic Acids Res, 32(Database issue), D452–D455.

Ispolatov, I., Yuryev, A., Mazo, I., and Maslov, S. (2005). Binding properties and evolution of homodimers in protein-protein interaction networks. Nucleic Acids Res, 33(11), 3629–3635.

Jeong, H., Tombor, B., Albert, R., Oltvai, Z. N., and Barabsi, A. L. (2000). The large-scale organization of metabolic networks. Nature, 407(6804), 651–654.

Light, S., Kraulis, P., and Elofsson, A. (2005). Preferential attachment in the evolution of metabolic networks. BMC Genomics, 6, 159.

Lukatsky, D. B., Shakhnovich, B. E., Mintseris, J., and Shakhnovich, E. I. (2007). Structural similarity enhances interaction propensity of proteins. J Mol Biol, 365(5), 1596–1606.

Pastor-Satorras, R., Smith, E., and Sol, R. V. (2003). Evolving protein interaction networks through gene duplication. J Theor Biol, 222(2), 199–210.

Pereira-Leal, J. B. and Teichmann, S. A. (2005). Novel specificities emerge by stepwise duplication of functional modules. Genome Res, 15(4), 552–559.

Peri, S., Navarro, J. D., Amanchy, R., Kristiansen, T. Z., Jonnalagadda, C. K., Surendranath, V., Niranjan, V., Muthusamy, B., Gandhi, T. K. B., Gronborg, M., Ibarrola, N., Deshpande, N., Shanker, K., Shivashankar, H. N., Rashmi, B. P., Ramya, M. A., Zhao, Z., Chandrika, K. N., Padma, N., Harsha, H. C., Yatish, A. J., Kavitha,M. P.,Menezes,M., Choudhury, D. R., Suresh, S., Ghosh, N., Saravana, R., Chandran, S., Krishna, S., Joy, M., Anand, S. K., Madavan, V., Joseph, A., Wong, G.W., Schiemann,W. P., Constantinescu, S. N., Huang, L., Khosravi-Far, R., Steen, H., Tewari, M., Ghaffari, S., Blobe, G. C., Dang, C. V., Garcia, J. G. N., Pevsner, J., Jensen, O. N., Roepstorff, P., Deshpande, K. S., Chinnaiyan, A. M., Hamosh, A., Chakravarti, A., and Pandey, A. (2003). Development of human protein reference database as an initial platform for approaching systems biology in humans. Genome Res, 13(10), 2363–2371.

Prince, V. E. and Pickett, F. B. (2002). Splitting pairs: the diverging fates of duplicated genes. Nat Rev Genet, 3(11), 827–837.

Saito, R., Suzuki, H., and Hayashizaki, Y. (2003). Construction of reliable protein-protein interaction networks with a new interaction generality measure. Bioinformatics, 19(6), 756–763.

Salwinski, L., Miller, C. S., Smith, A. J., Pettit, F. K., Bowie, J. U., and Eisenberg, D. (2004). The database of interacting proteins: 2004 update. Nucleic Acids Res, 32(Database issue), D449–D451.

Straßer, W., Siegl, D., Önder, K., and Bauer, J. (2006). Insilico proteomics system: Integration and application of protein and protein-protein interaction data using microsoft .net. Journal of Integrative Bioinformatics, 3(2).

Tan, S.-H., Zhang, Z., and Ng, S.-K. (2004). Advice: Automated detection and validation of interaction by co-evolution. Nucleic Acids Res, 32(Web Server issue), W69–W72.

Teichmann, S. A. and Babu, M. M. (2004). Gene regulatory network growth by duplication. Nat Genet, 36(5), 492–496.

Tirosh, I. and Barkai, N. (2005). Computational verification of protein-protein interactions by orthologous co-expression. BMC Bioinformatics, 6, 40.

van Noort, V., Snel, B., and Huynen, M. A. (2004). The yeast coexpression network has a small-world, scale-free architecture and can be explained by a simple model. EMBO Rep, 5(3), 280–284.

Vàzquez, A., Flammini, A., Maritan, A., and Vespignani, A. (2003). Modeling of protein interaction networks. Complexus, 1, 38–44.

Yook, S.-H., Oltvai, Z. N., and Barabsi, A.-L. (2004). Functional and topological characterization of protein interaction networks. Proteomics, 4(4), 928–942.
